# Key role of fluorescence quantum yield in Nile Red staining method for determining intracellular lipids in yeast strains

**DOI:** 10.1186/s13068-022-02135-9

**Published:** 2022-04-15

**Authors:** Sergio Morales-Palomo, Marta Liras, Cristina González-Fernández, Elia Tomás-Pejó

**Affiliations:** 1 Biotechnological Processes Unit, IMDEA Energy, Avda. Ramón de la Sagra 3, 28935 Móstoles, Spain; 2 Photoactivated Processes Unit, IMDEA Energy, Avda. Ramón de la Sagra 3, 28935 Móstoles, Spain

**Keywords:** Fluorescence intensity, Gravimetric extraction, Lipid quantification, Nile Red, Fluorescence quantum yield, Oleaginous yeast

## Abstract

**Background:**

Microbial lipids are found to be an interesting green alternative to expand available oil sources for the chemical industry. Yeasts are considered a promising platform for sustainable lipid production. Remarkably, some oleaginous yeasts have even shown the ability to grow and accumulate lipids using unusual carbon sources derived from organic wastes, such as volatile fatty acids. Recent research efforts have been focused on developing rapid and accurate fluorometric methods for the quantification of intracellular yeast lipids. Nevertheless, the current methods are often tedious and/or exhibit low reproducibility.

**Results:**

This work evaluated the reliability of different fluorescence measurements (fluorescence intensity, total area and fluorescence quantum yield) using Nile Red as lipid dye in two yeast strains (*Yarrowia lipolytica* ACA-DC 50109 and *Cutaneotrichosporon curvatum* NRRL-Y-1511). Different standard curves were obtained for each yeast specie. Fermentation tests were carried with 6-month difference to evaluate the effect of the fluorometer lamp lifetime on lipid quantification.

**Conclusions:**

Fluorescence quantum yield presented the most consistent measurements along time and the closer estimations when compared with lipids obtained by conventional methods (extraction and gravimetrical determination). The need of using fluorescence quantum yield to estimate intracellular lipids, which is not the common trend in studies focused on microbial lipid production, was stressed. The information here provided will surely enable more accurate results comparison.

**Supplementary Information:**

The online version contains supplementary material available at 10.1186/s13068-022-02135-9.

## Background

The use of vegetable oils as raw materials is not fulfilling the increasing demand of oleochemicals. Thus, microbial oils are envisaged as interesting green alternatives to expand available oil sources for the chemical industry. Yeasts stand out as the most promising microorganisms in terms of microbial oils production since they can accumulate up to 80% of lipids per dry weight in their cells [1]. Moreover, they present high growth rates, short life cycles and versatility when it comes to utilization of different substrates [2]. High lipid contents have been achieved in yeasts using sugars as carbon sources [3]. However, the use of glucose represents 60% to 80% of the overall production cost [4]. Thus, in the last years, research efforts have been focused to increase lipid production in yeasts using low-cost carbon sources derived from organic residues, such as volatile fatty acids (VFAs) [5]. VFAs are organic acids regarded in the chemical industry as building blocks in the so-called carboxylate platform [6]. These acids can be sustainably produced after the hydrolytic and the acidogenic stages of anaerobic fermentation of organic wastes. To this end, optimum performing strains are intensively searched in the context of microbial oil accumulation from wastes. However, bioprospecting yeasts for lipids accumulation by means of conventional methods [7, 8] might be tedious, time consuming, reagent demanding and not environmentally friendly. To avoid these drawbacks, fluorometric methods have been proposed as feasible methods to measure lipids and easily identify strains with the ability to accumulate them [9, 10]. Among the various fluorescent dyes, Nile Red has been widely applied given its high specificity for microbial neutral lipids determination, high stability and low cost [11, 12]. However, this method exhibits low reproducibility as stated in previous reports [13, 14]. Presumably, the non-reproducible values attained with this dye rely on the fact that most of the studies report their results in relative fluorescence units, such as fluorescence intensity [15, 16]. The fluorescence intensity, measured in arbitrary units, depends on the fluorometer used and the equipment lamp lifetime, giving rise to inconsistent measurements that hinder results comparison [14]. Despite the importance of fluorescence quantum yield (*ф*_fl_) in fluorescence spectroscopy [14], this parameter is often neglected in most of the biotechnological reports dealing with lipids accumulation in yeasts. In fact, the *ф*_fl_ is the most important parameter for the characterization of fluorescence materials, reaction pathways and probes behavior [17]. The International Unit of Pure and Applied Chemistry (IUPAC) defines *ф*_fl_ as the number of photons emitted by fluorescence pathway per photons absorbed by the system [18]. *ф*_fl_ is an absolute number that expresses the probability of emission of a given system and it is, therefore, a physical characteristic of a substance independent of external factors such a lamp lifetime, lamp intensity or fluorometer used.

This systematic investigation was designed to underpin the key role of *ф*_fl_. To this end, the reliability of different fluorescence measurements (*i.e.* fluorescence emission intensity (*I*_E_), total emission area (*A*_E_) and *ф*_fl_) for direct estimation of yeast lipid content using Nile Red was evaluated. To unravel the effect of lamp lifetime on lipid determination based on the most common fluorescence measurements, fermentations tests were carried with 6-month time span to build standard curves using different carbon sources (yeast extract peptone dextrose medium (YPD) and VFA-rich synthetic media). Additionally, the reliability of each fluorescence measurement was confirmed by comparison with the values attained when conducting lipids determination by gravimetric analysis.

## Results and discussion

### Standard curve’s reliability along time

Yeast lipid content at 24 h of fermentation in *Y. lipolytica* ACA-DC 50109 and *C. curvatum* NRRL-Y-1511 grown in YPD and VFAs-synthetic media was determined gravimetrically and by fluorescence to build different standard curves with 6-month difference. Each cell sample was diluted in phosphate buffer solution (PBS) to different OD_600_ (0.2, 0.4, 0.6, 0.8 and 1.0) for attaining different lipid content. By doing so, an accurate linear relationship between the fluorescence determination and the lipids determined gravimetrically was built. Cell samples collected at the same fermentation time were subjected to fluorescence analysis by staining cells Nile Red and *I*_E,_ *A*_E_ and *ф*_fl_ were determined.

**Fig. 1:**
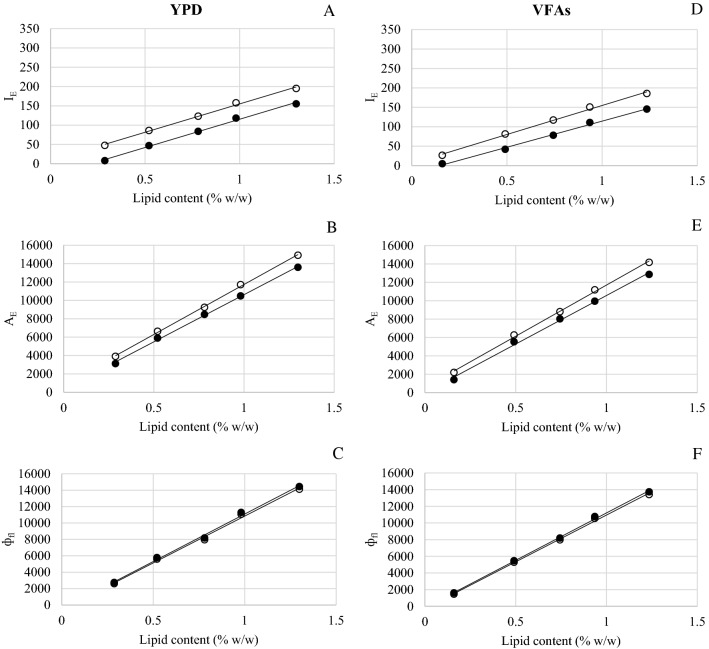
Standard curves obtained using *I*_E_ (fluorescence emission intensity) (**A**, **D**), *A*_E_ (fluorescence emission area) (**B**, **E**) and *ф*_fl_ (fluorescence quantum yield) (**C**, **F**) from *C. curvatum* NRRL-Y-1511 grown in YPD and VFAs (○: month-1, ●: month-7), respectively

**Fig. 2:**
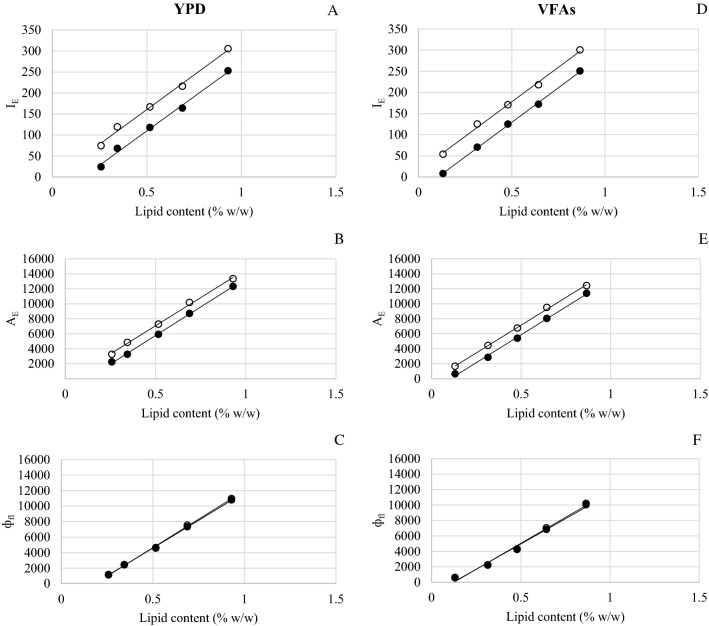
Standard curves obtained using *I*_E_ (fluorescence emission intensity) (**A**, **D**), *A*_E_ (fluorescence emission area) (**B**, **E**) and *ф*_fl_ (fluorescence quantum yield) (**C**, **F**) from *Y. lipolytica* ACA-DC 50109 grown in YPD and VFAs (○: month-1, ●: month-7), respectively

Figures 1 and 2 show the standard curves for lipid quantification in *C. curvatum* NRRL-Y-1511 and *Y. lipolytica* ACA-DC 50,109 grown on YPD and VFAs-rich media in month-1 and month-7. As expected, no significant lipid production was detected when using *Sacharomyces cerevisiae* Ethanol Red™ strain in YPD media, indicating that this methodology was not affected by false positives. Although both *C. curvatum* NRRL-Y-1511 and *Y. lipolytica* ACA-DC 50109 are well-known oleaginous yeasts, standard curves obtained by the different lipid quantification methods (*I*_E_, *A*_E_ and *ф*_fl_) differed. In principle, these differences could be due to their different cell membranes and lipid composition between yeast families [19]. Lipids surrounding membrane proteins are not only crucial for membrane structure and function but they might affect the physical properties of the membrane matrix (*e.g*. fluidity, thickness,…) [9, 20, 21]. The different lipids in biological membranes could affect Nile Red penetration through the yeast membrane and its subsequent interaction with intracellular lipids. In this sense, previous reports have concluded that some yeast and microalgae strains require dimethyl sulfoxide (DMSO) as a carrier for Nile Red to penetrate the cells [15, 16, 22]. Despite increasing the fluorescence intensity, the incubation time of Nile Red using DMSO can last 60 min [23]. This value is particularly long when compared to the 5 min required when using PBS (in this study). This suggests that there is no universal method for lipid determination in all microorganisms but methods should be specifically tailored to the particular oleaginous microbial system. According to this, and in view of the data presented in Figs. 1 and 2, a standard curve should be stablished for each yeast species when determining lipid content by fluorometric means.

In all cases, and independently of the fluorescence parameters employed to measure lipid content, the standard curves obtained obeyed a linear regression. More specifically, the correlation coefficient (*R*^2^) reached values over 0.98 in all standard curves (Additional file 1: Table S1). Regarding the *I*_E_ (Figs. 1A, D and  2A, D for *C. curvatum* NRRL-Y-1511 and *Y. lipolytica* ACA-DC 50109, respectively), lower R^2^ values were achieved in previous studies when a correlation between fluorometric and gravimetric methods was stablished using these two yeast strains for lipid quantification (0.91 and 0.92, [16, 24], respectively). Moreover, the linear regression curves based on *I*_E_ were completely different to the ones obtained herein. The reasons for those differences might be attributed to the different fluorometers used and/or the lifetime of the lamps. In this sense, Figs. 1A, D, 2A and D revealed a difference when comparing curves obtained at month-1 and month-7 with both yeast strains. This corroborated the hypothesis that the lamp lifetime affected the lipid estimation, providing inconsistent and non-reproducible measurements over time. These results were in good agreement with what De la Hoz Siegler and co-workers [13] observed after determining the correlation between *I*_E_ and lipid content (% w/w) using four different microalgae strains. Even if the correlation coefficient was greater than 0.99 in all cases, this investigation reported that each standard curve was specific for the fluorometer, sets of filters and age of the lamp used. Thus, a recalibration of the standard curve was recommended for each experiment to verify the linearity of the relationship between *I*_E_ and lipid content. To circumvent specific equipment features affecting the fluorescence results, it is necessary to rely on absolute responses rather than relative ones. To this end, *ф*_fl_ was proposed as a reliable parameter for the determination of lipid content in yeast (and any other microbial system). As observed in Figs. 1C, F and 2C, F for *C. curvatum* NRRL-Y-1511 and *Y. lipolytica* ACA-DC 50109, respectively, the standard curves obtained with *ф*_fl_ presented the greatest stability and reliability because *ф*_fl_ was not only governed by the linear equation, but by the conversion from *A*_E_ to *ф*_fl_ (as it can be seen in Eq. 1) which helps to minimize the analytical error. These findings underpinned the key role of *ф*_fl_ as an appropriate fluorescence measurement when determining microbial lipid content. By using *ф*_fl_ for lipid quantification, the lamp lifetime and differences in equipment can be disregarded. It can be thus stated that lipid determination via fluorescence should be based on *ф*_fl_, and not on other fluorescence parameters that can lead to inaccurate values. In this sense, the *ф*_fl_ standard curves provided in this article could be employed by other researchers using the same yeast strains.

**Fig. 3:**
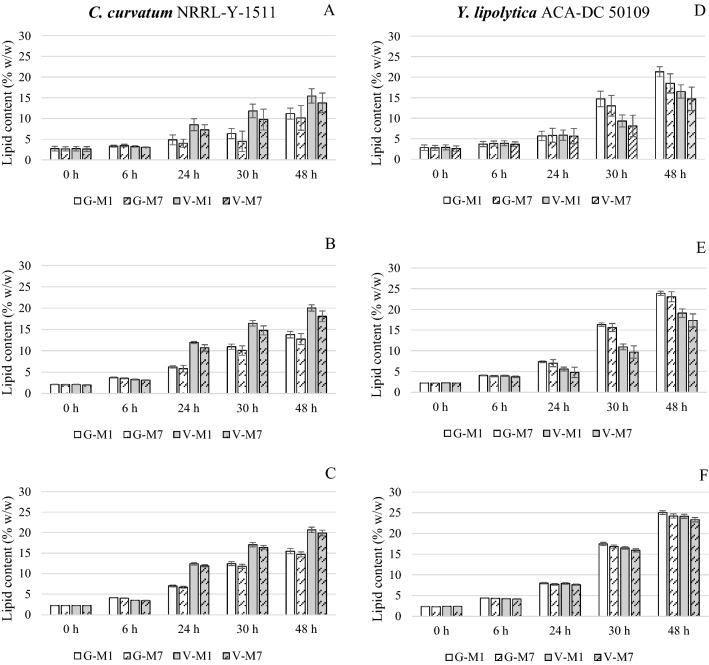
Lipid content (% w/w) in cells from fermentations performed in month-1 and month-7 estimated using standard curves obtained at month-1 (M1) and month-7 (M7), respectively. Curves based on *I*_E_ (fluorescence emission intensity) (**A**, **D**), *A*_E_ (fluorescence emission area) (**B**, **E**) and *ф*_fl_ (fluorescence quantum yield) (**C**, **F**). *G: lipids in cells grown in glucose media; *V: lipids in cells grown in VFAs-rich media

**Fig. 4:**
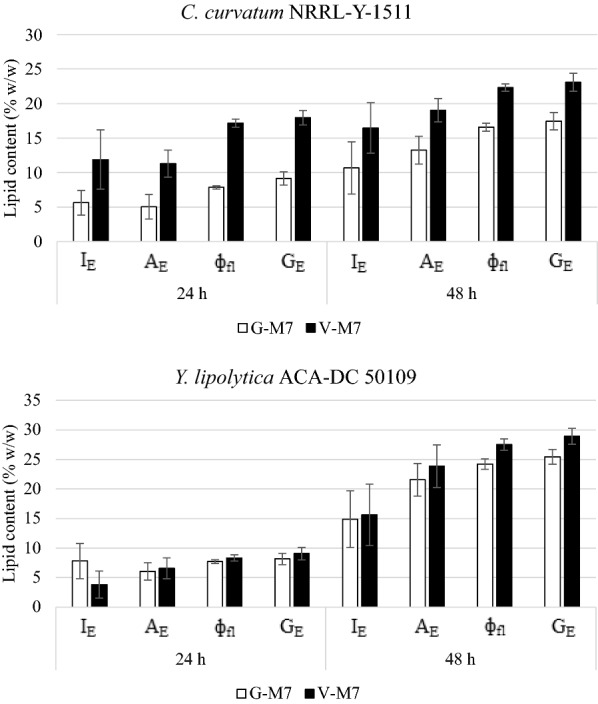
Lipid content (% w/w) in cells from fermentations performed in month-7 estimated using standard curve obtained at month-7 (M7) (*I_E_: fluorescence emission intensity; *A_E_: fluorescence emission area; *ф_fl_: fluorescence quantum yield; *G_E_: gravimetric extraction; *G: lipids in cells grown in glucose media; *V: lipids in cells grown in VFAs-rich media)

### Lipid quantification with different standard curves

Lipid content in cell samples obtained in month-1 or month-7 with *C. curvatum* NRRL-Y-1511 or *Y. lipolytica* ACA-DC 50109 and glucose or VFAs as carbon source was quantified using the standard curve built at the same month (*i.e.* cell samples from month-1 fermentation were analyzed with month-1 standard curves and cell samples from month-7 fermentation were analyzed with month-7 standard curves). As shown in Fig. 3A and D, there were no significant differences (*p* > 0.05) in the lipid content (% w/w) using *I*_E_-based standard curves when fermentations were performed with 6-month difference regardless the yeast species and the carbon source (glucose or VFAs). However, the clear increase in error bars in lipid content estimated with month-7 curve when compared to month-1 (significant differences in variances (*p* < 0.05)), corroborated that the lamp lifetime affected the measurement giving rise to higher analytical error. The same tendency was observed when using *A*_E_-based standard curves for lipid quantification (Fig. 3B, E). Remarkably, when using *ф*_fl,_ the error bars in the lipid content quantification were significantly reduced (Fig. 3C, F). Once again, these results pointed out to *ф*_fl_ as a consistent and reliable parameter for lipids quantification in yeasts. The fact that no differences were observed on lipid estimation when using glucose or VFAs standard curves highlighted as well the appropriateness of this methodology despite the carbon sources employed in the fermentations. Given the similarities of VFAs with fatty acids, a possible interaction of VFAs with Nile Red has been reported [25, 26]. Therefore, in order to avoid false fluorescence signals when using VFAs medium together with Nile Red, it is highly recommended to conduct a throughout wash of the samples. Different culture media can affect inherent features of yeast cells (cell morphology, lipid droplet size or cell wall thickness) [27]. However, as it can be seen in Fig. 3, no remarkable differences were observed despite the different carbon sources employed for yeast cultivation. Thus, the feasibility of this methodology was confirmed for lipid determination from different carbon sources. Many researchers have tried to optimize the fluorometric method by investigating the effect of biomass concentration, use of carriers for dyes penetration or the use of different staining dyes, concentration or incubation time [13, 28, 29]. Results are always displayed in terms of relative units, while the solution to avoid inconsistent measurement would be the use of fluorimeter absolute values, as the *ф*_fl_ provides.

### Correlation between Nile Red and gravimetric method for lipid content determination in yeasts

To validate the use of the proposed method based on *ф*_fl_, a comparison of lipid content determination using gravimetric and fluorometric methods was performed. Lipids from *C. curvatum* NRRL-Y-1511 and *Y. lipolytica* ACA-DC 50109, obtained from fermentations performed in month-7 in both YPD and VFAs-synthetic media, were extracted after 24 h and 48 h of fermentation and their content (% w/w) was gravimetrically determined. The different fluorescence parameters (*I*_E_, *A*_E_ and *ф*_fl_) were also measured to determine lipid content in cells from the same fermentation sampling times (24–48 h). Since no differences in the *ф*_fl_-based standard curves obtained with glucose and VFAs were observed (Fig. 1C, F and 2C, F), month-7 standard curves obtained using the media with VFAs were used to process all fluorometric data. As shown in Fig. 4, lipid content obtained with *I*_E_, which is the most common method used when conducting this type of yeast screenings [23, 29], presented the highest error bars. Furthermore, significant differences (*p* < 0.05) were observed in the lipids content obtained when using the gravimetric method and *I*_E_. This later mismatch can be seen in the data collected with both yeast species independently on the carbon source (glucose or VFAs) at 48 h (Fig. 4). In the case of *C. curvatum* NRRL-Y-1511 (Fig. 4), the differences in terms of lipid content were even more significant when *I*_E_ and *A*_E_ were compared with the gravimetrically determined lipids at 24 h. Regardless of the yeast species and carbon sources employed for the fermentations, *ф*_fl_ provided the most consistent measurements and presented greater correlations with the lipid content gravimetrically obtained (no significant differences in means were observed at 24 h and 48 h of fermentation (*p* > 0.05)).

These results, together with those mentioned in the above sections, supported the need of using *ф*_fl_ when standard fluorescence methods based on Nile Red are employed to estimate intracellular yeast lipids. The use of *ф*_fl_ enabled the closest correlation between the lipids content determined by fluorescence and conventional gravimetry.

Lipid contents of 43.4% w/w [30] and > 70% w/w (unpublished results) were achieved with the same *Y. lipolytica* ACA-DC 50109 strain and determined with the methodology described herein. These facts indicated that the *ф*_fl_ could also be used to quantify large amounts of lipids not being affected by possible saturations.

## Conclusions

In this investigation, *ф*_fl_ was demonstrated to be a key factor when determining lipid content in yeasts. Lipid content in two different yeast strains (*C. curvatum* NRRL-Y-1511 and *Y. lipolytica* ACA-DC 50109), grown on different carbon sources (glucose and VFAs), was successfully determined using *ф*_fl_. Unlike other protocols based on *I*_E_, robust and consistent linear *ф*_fl_ standard curves were stablished for each yeast strain, which can be used in other investigations. The *ф*_fl_ protocol can be reproduced in any laboratory since the measurements do not depend on external factors. The use of *ф*_fl_ provided equal lipid content to that obtained after gravimetry determination. This investigation will for sure enable scientists to better compare results.

## Methods

### Oleaginous yeast strains and preinoculum preparation

*Yarrowia lipolytica* ACA-DC 50109 (culture collection of Agricultural University of Athens) and *Cutaneotrichosporon curvatum* NRRL Y-1511 (previously known as *Cryptococcus curvatus* and recently also named as *Cutaneotrichosporon oleaginosus*) were used for lipid production and determination using different carbon sources, namely VFAs and glucose. *Saccharomyces cerevisiae* Ethanol Red™ was used as a negative control. *S. cerevisiae* was only used with 20 g/L of glucose because it is not able to use VFAs as carbon source. Yeast strains were kept in glycerol 30% (v/v) at -80 °C. Yeasts were grown on YPD agar plates (10 g/L yeast extract, 20 g/L peptone, 20 g/L glucose and 20 g/L agar). Pre-cultures were performed by inoculating a colony of the specific strain in YPD liquid medium (same composition as mentioned above, except for the agar) and incubated overnight in a rotary shaker at 170 rpm and 27 ºC, until the culture reached the exponential growth phase.

### Fermentation conditions

100 mL of YPD liquid medium or VFAs-synthetic medium were used as fermentation media. VFAs medium contained 10.3 g/L acetic acid, 0.2 g/L propionic acid, 2.7 g/L butyric acid, 0.3 g/L valeric acid and 1.8 g/L hexanoic acid, mimicking the VFAs profile and concentrations exhibited in real digestates obtained from the anaerobic fermentation of food waste [31].

All fermentations were inoculated with yeast cells to reach an optical density at 600 nm (OD_600_) of 1. Triplicates of each experiment were carried out in 250-mL Erlenmeyer flasks with baffles at the bottom to promote aeration. Each flask contained 100 mL of fermentation medium. Initial pH was set at 6.0 using NaOH 3 M and not further adjusted during fermentation. Fermentations were performed in a rotary shaker at 170 rpm and 27 ºC until 95–100% carbon source (*i.e.* glucose or VFAs) was consumed. 1-mL samples were taken periodically from 0 h to 48 h of fermentation to analyze glucose and VFAs consumption, cell growth and lipid content.

The first batch of experiments with both yeast strains (*Y. lipolytica* ACA-DC 50109 and *C. curvatum* NRRL-Y-1511) and both fermentation media (YPD and VFAs-synthetic media) was performed in month-1. To determine the effect of lamp lifetime, experiments were repeated in month-7 under exactly the same conditions. It is worth mentioning that the lamp was actively used for 70 h/month during this period of time.

### Growth and biomass determination

Yeast growth was evaluated by measuring OD_600_ of the cultures (Spectroquant® Pharo 100 spectrophotometer). For dry weight determination, 5 mL of culture was filtered through a pre-weighted 0.45 μm glass fiber membrane (Millipore, MA, USA) and dried at 105 ºC until constant weight [32]. All lipid contents reported herein are in terms of biomass dry weight. In this manner, the mismatch associated to the different cell counts present when assayed at the same OD can be disregarded [21].

### Analytical methods

VFAs and glucose were determined by liquid chromatography with an Agilent 1260 HPLC-RID (Agilent, Santa Clara, CA, USA) equipped with a Cation H Refill Cartridge Microguard column (Biorad, Hercules, CA, USA) and an Aminex HPX- 87H ion exclusion column (300 × 7.8 mm I.D.) (Biorad). The mobile phase was 5 mM H_2_SO_4_ and elution was performed in isocratic mode at a flow rate of 0.6 mL/min for VFAs and 0.5 mL/min for glucose. The injected sample volume was 20 μL. The oven and detector were set at 25 °C and 35 °C, respectively.

### Fluorescence spectroscopy methods

#### Nile Red staining procedure

**Fig. 5:**
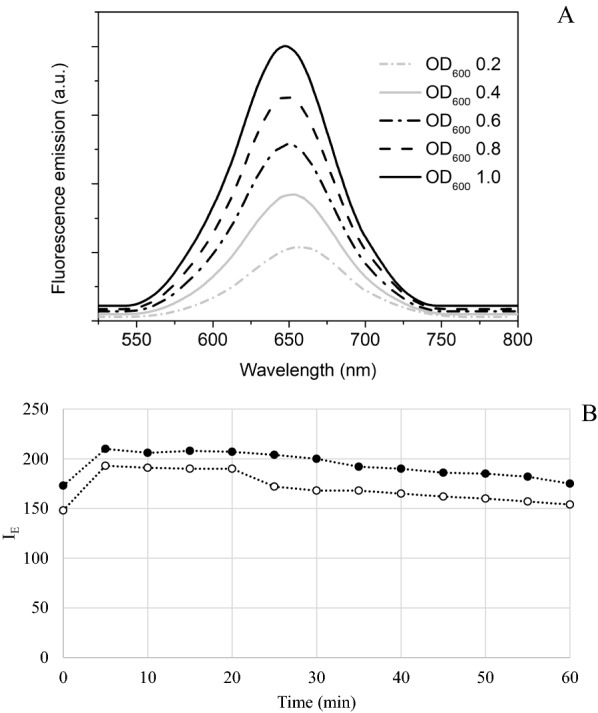
Fluorescence emission of *C. curvatum* NRRL-Y-1511 cells stained with Nile Red with excitation at 488 nm at an OD_600_ of 0.2, 0.4, 0.6, 0.8 and 1.0 (**A**); *I*_E_ of *Y. lipolytica* ACA-DC 50109 (●) and *C. curvatum* NRRL-Y-1511 (○) cell broth stained with Nile Red at different incubation times (**B**)

Nile Red (9-diethylamino-5H-benzo[a]phenoxazine-5-one, Acros Organics) was dissolved in acetone to prepare a stock solution at 1 mg/mL. Cells were collected from fermentation broths by centrifugation (5000 rpm, 5 min) (Heraeus, Megafuge16, Thermo Scientific) and washed twice with 0.9% NaCl solution. Afterwards, the suspended cells in PBS were pretreated at 50 ºC in rotary shaker for 20 min and then cooled down to room temperature. After pretreatment, the cell suspensions were stained with Nile Red at a final concentration of 1 µg/mL. The stained samples were kept 5 min in darkness and subjected to fluorescence determination, since this time was selected as the most efficient staining period to achieve maximum fluorescence (Fig. 5B). Cells samples taken at 24 h were used for building the standard curves. In this case, cell pellets were suspended and diluted to different OD_600_ (0.2, 0.4, 0.6, 0.8 and 1.0) using PBS where different fluorescence emissions corresponded to different OD_600_ of the cell sample (Fig. 5A). Lipid content in cells diluted at different OD_600_ was gravimetrically determined as explained below.

#### Fluorescence determination

Three parameters related with fluorescence determination were evaluated: (i) intensity of fluorescence emission (*I*_E_) as the number of counts in the maximum of the fluorescence (*ca*. 570 nm) of each spectrum, (ii) fluorescence emission area (*A*_E_) as the area below each spectrum curve and, (iii) fluorescence emission quantum yield (*ф*_fl_).

Two methods can be followed for *ф*_fl_ determination: (i) collecting the whole spatially distributed fluorescence emission or reconstructing this spatial profile by means of an integrating sphere detector (which is not always available in the commercial fluorometers) and (ii) comparative method that relies on the previous knowledge of the *ф*_fl_ of some standard dyes in determined solvents. The second one is most standardized method since it only requires simple light absorption and fluorescence measurements with conventional instrumentation. For this reason, this latter method was selected to calculate the *ф*_fl_ in this investigation.

**Fig. 6:**
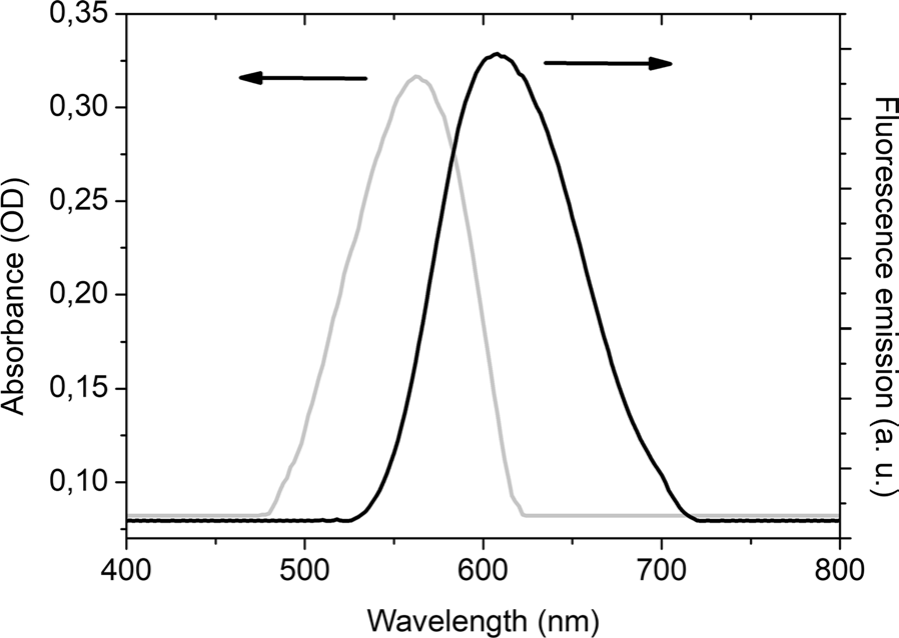
Absorbance and fluorescence emission spectra of *C. curvatum* NRRL-Y-1511 cells stained with Nile Red obtained at an exciting wavelength of 488 nm

The light absorption at the exciting wavelength (*ca.* 488 nm; OD_488 nm_) of standard solution without cells and stained Nile Red cell suspension were determined by means of a Spectroquant® Pharo 100 spectrophotometer. The fluorescence emission intensity of both solutions, standard (*I*_E(s)_) and stained Nile Red cell suspension (*I*_E_), was determined by means of PerkinElmer® LS 55 Fluorescence Spectrometer as the number of counts in the maximum of each fluorescence spectrum excited at 488 nm. The area of both standard solution (*A*_E(S)_) and stained Nile Red cell suspension (*A*_E_) were determined mathematically by integrating their respective emission spectrum. As an example, Fig. 6 represents the absorbance and fluorescence emission spectra of *C. curvatum* NRRL-Y-1511 cells stained with Nile Red and obtained at an exciting wavelength of 488 nm.

The *ф*_fl_ is calculated by employing Eq. 1:

1 $${\phi }_{fl}=\frac{{I}_{\mathrm{A}\left(\mathrm{s}\right)}{A}_{\mathrm{E}}{\eta }^{2}}{{I}_{\mathrm{A}}{A}_{\mathrm{E}\left(\mathrm{s}\right)}{\eta (s)}^{2}}\phi (s)$$

where ${I}_{A}=1-{10}^{-{OD}_{488 nm}}$; *A*_E_ fluorescence emission area; η refractive index of each solvent and *ф*(s) the quantum yield of the standard solution (Nile Red dissolved in acetone *ф*_fl_ = 0.32, [33]).

### Lipid extraction and gravimetric determination

Total lipid extraction was conducted using the method described by Folch [8] with some modifications [34]. Briefly, cell biomass (0.4 g/L) was centrifuged at 5000 rpm for 10 min at 4 ºC (Heraeus, Megafuge16, Thermo Scientific) and dried at 105 ºC overnight. Dry biomass (around 0.2 g dry weight) was mixed with a solution of chloroform and methanol (2:1 v/v) under reflux for 4 h. The extract was filtered (Watman N.1 paper) and 0.88% KCl solution was used to wash the organic phase. Samples were dried using anhydrous Na_2_SO_4_ (Sigma). The chloroform phase containing the lipids was evaporated using a Rotavapor R-215 (BUCHI) at 40 ºC and 350 mb of vacuum. Finally, total cellular lipids were gravimetrically determined and expressed as grams of lipid per grams of dry biomass (% w/w).

A parametric one-way ANOVA and F-test were used for the assessment of means and variances of microbial lipid content (% w/w) (confidence interval 90%), respectively. Differences were considered significant at *p*-value < 0.05.

## Supplementary Information

**Additional file 1: Table S1**. R^2^ and standard curves equations obtained with *Cutaneotrichosporon curvatum* NRRL-Y-1511 and *Yarrowia lipolytica* ACA-DC 50109.

## Data Availability

All data generated or analysed during this study are included in this published article.

## References

[1] Juanssilfero AB, Kahar P, Amza RL, Miyamoto N, Otsuka H, Matsumoto H (2018). Effect of inoculum size on single-cell oil production from glucose and xylose using oleaginous yeast *Lipomyces starkeyi*. J Biosci Bioeng.

[2] Tomás-Pejó E, Morales-Palomo S, González-Fernández C (2021). Microbial lipids from organic wastes: outlook and challenges. Bioresour Technol.

[3] Carsanba E, Papanikolaou S, Fickers P, Erten H (2020). Lipids by *Yarrowia lipolytica* strains cultivated on glucose in batch cultures. Microorganisms.

[4] Patel A, Mikes F, Matsakas L (2018). An overview of current pretreatment methods used to improve lipid extraction from oleaginous microorganisms. Molecules.

[5] Llamas M, Dourou M, González-Fernández C, Aggelis G, Tomás-Pejó E (2020). Screening of oleaginous yeasts for lipid production using volatile fatty acids as substrate. Biomass Bioenerg.

[6] Llamas M, Magdalena JA, González-Fernández C, Tomás-Pejó E (2020). Volatile fatty acids as novel building blocks for oil-based chemistry via oleaginous yeast fermentation. Biotechnol Bioeng.

[7] Bligh EG, Dyer WJ (1959). A rapid method of total lipid extraction and purification. Can J Biochem Physiol.

[8] Folch J, Lees M, Sloane Stanley GH (1956). A simple method for the isolation and purification of total lipidies from animal tissues. J Biol Chem.

[9] Poli JS, Lützhøft HCH, Karakashev DB, Valente P, Angelidaki I (2014). An environmentally-friendly fluorescent method for quantification of lipid contents in yeast. Biores Technol.

[10] Zhao C, Luo MT, Huang C, Chen XF, Xiong L, Li HL (2019). Determining intracellular lipid content of different oleaginous yeasts by one simple and accurate Nile Red fluorescent method. Prep Biochem Biotechnol.

[11] Brown WJ, Sullivan TR, Greenspan P (1992). Nile Red staining of lysosomal phospholipid inclusions. Histochemistry.

[12] Cooksey KE, Guckert JB, Williams SA, Callis PR (1987). Fluorometric determination of the neutral lipid content of microalgal cells using Nile Red. J Microbiol Methods.

[13] De la Hoz SH, Ayidzoe W, Ben-Zvi A, Burrell RE, McCaffrey WC (2012). Improving the reliability of fluorescence-based neutral lipid content measurements in microalgal cultures. Algal Res.

[14] Lakowicz JR (2006). Principles of fluorescence spectroscopy.

[15] Sitepu IR, Ignatia L, Franz AK, Wong DM, Faulina SA, Tsui M (2012). An improved high-throughput Nile Red fluorescence assay for estimating intracellular lipids in a variety of yeast species. J Microbiol Methods.

[16] Miranda C, Bettencourt S, Pozdniakova T, Pereira J, Sampaio P, Franco-Duarte R (2020). Modified high-throughput Nile Red fluorescence assay for the rapid screening of oleaginous yeasts using acetic acid as carbon source. BMC Microbiol.

[17] Demchenko AP (2009). Introduction to fluorescence sensing.

[18] Braslavsky SE (2007). Glossary of terms used in photochemistry 3rd edition: (IUPAC Recommendations 2006). Pure Appl Chem.

[19] Renne MF, de Kroon AIPM (2018). The role of phospholipid molecular species in determining the physical properties of yeast membranes. FEBS Lett.

[20] Dowhan W, Bogdanov M, Mileykovskaya E (2008). Functional roles of lipids in membranes. Biochemistry of lipids, lipoproteins and membranes.

[21] Hicks RH, Chuck CJ, Scott RJ, Leak DJ, Henk DA (2019). Comparison of Nile Red and cell size analysis for high-throughput lipid estimation within oleaginous yeast. Eur J Lipid Sci Technol.

[22] Chen W, Sommerfeld M, Hu Q (2011). Microwave-assisted Nile Red method for in vivo quantification of neutral lipids in microalgae. Biores Technol.

[23] Ramírez-Castrillón M, Jaramillo-Garcia VP, Barros HL, Pêgas Henriques JA, Stefani V, Valente P (2020). Dataset of Nile Red fluorescence readings with different yeast strains, solvents, and incubation times. Data.

[24] Kimura K, Yamaoka M, Kamisaka Y (2004). Rapid estimation of lipids in oleaginous fungi and yeasts using Nile Red fluorescence. J Microbiol Methods.

[25] Greenspan P, Mayer EP, Fowler SD (1985). Nile Red: a selective fluorescent stain for intracellular lipid droplets. J Cell Biol.

[26] Güney M, Oz AT, Kafkas E (2015). Comparison of lipids, fatty acids and volatile compounds of various kumquat species using HS/GC/MS/FID techniques. J Sci Food Agric.

[27] Ribeiro RA, Vitorino MV, Godinho CP, Bourbon-Melo N, Robalo TT, Fernandes F (2021). Yeast adaptive response to acetic acid stress involves structural alterations and increased stiffness of the cell wall. Sci Rep.

[28] Zhao C, Yao QS, Wang C, Luo MT, Huang C, Chen XF (2021). Fast measurement of lipid content of oleaginous yeast trichosporon dermatis cultured in lignocellulosic hydrolysates using fluorescent method. J Chem Soc Pak.

[29] Ramírez-Castrillón M, Jaramillo-Garcia VP, Lopes Barros H, Pegas Henriques JA, Stefani V, Valente P (2021). Nile Red incubation time before reading fluorescence greatly influences the yeast neutral lipids quantification. Front Microbiol.

[30] Morales-Palomo S, González-Fernández C, Tomás-Pejó E (2022). Prevailing acid determines the efficiency of oleaginous fermentation from volatile fatty acids. J Environ Chem Eng.

[31] Greses S, Tomás-Pejó E, Gónzalez-Fernández C (2020). Agroindustrial waste as a resource for volatile fatty acids production via anaerobic fermentation. Biores Technol.

[32] Chanlett ET (1947). Standard methods for the examination of Water and sewage. Am J Public Health Nations Health.

[33] Dutta AK, Kamada K, Ohta K (1996). Spectroscopic studies of Nile Red in organic solvents and polymers. J Photochem Photobiol A.

[34] Dourou M, Mizerakis P, Papanikolaou S, Aggelis G (2017). Storage lipid and polysaccharide metabolism in *Yarrowia lipolytica* and *Umbelopsis isabellina*. Appl Microbiol Biotechnol.

