# Supplementary material for: Key role of fluorescence quantum yield in Nile Red staining method for determining intracellular lipids in yeast strains
Source: Biotechnol Biofuels Bioprod. 2022 Apr 15;15:37. doi: 10.1186/s13068-022-02135-9 (PMC9019942; doi:10.1186/s13068-022-02135-9)
Supplement: Supplementary file 1 — Additional file 1: Table S1. R2 and standard curves equations obtained with Cutaneotrichosporon curvatum NRRL-Y-1511 and Yarrowia lipolytica ACA-DC 50109. [file 13068_2022_2135_MOESM1_ESM.docx]

**Table S1.** R^2^ and standard curves equations obtained with *Cutaneotrichosporon curvatum* NRRL-Y-1511 and *Yarrowia lipolytica* ACA-DC 50109*.*

| Yeast strain | Carbon source | Month | Fluorescence measurement | R^2^ | Equation |
| --- | --- | --- | --- | --- | --- |
| *C. curvatum* | Glucose | M1 | I_E_ | 0.9958 | y = 146.96x + 7.9691 |
|  |  |  | A_E_ | 0.999 | y = 10877x + 852.91 |
|  |  |  | ф_fl_ | 0.9947 | y = 11421x – 577.11 |
| *C. curvatum* | Glucose | M7 | I_E_ | 0.9954 | y = 146.59 x – 31.3 |
|  |  |  | A_E_ | 0.9986 | y = 10263x + 357.41 |
|  |  |  | ф_fl_ | 0.9952 | y = 11375x – 480.07 |
| *C. curvatum* | VFAs | M1 | I_E_ | 0.9959 | y = 149.09x + 5.624 |
|  |  |  | A_E_ | 0.9984 | y = 11139x + 562.57 |
|  |  |  | ф_fl_ | 0.9986 | y = 12384x – 172.5 |
| *C. curvatum* | VFAs | M7 | I_E_ | 0.9954 | y = 134.24x – 19.736 |
|  |  |  | A_E_ | 0.9963 | y = 10453x – 1080.8 |
|  |  |  | ф_fl_ | 0.9981 | y = 12725x – 57.845 |
| *Y. lipolytica* | Glucose | M1 | I_E_ | 0.9938 | y = 329.81– 4.3204 |
|  |  |  | A_E_ | 0.9977 | y = 15006x – 434.52 |
|  |  |  | ф_fl_ | 0.9988 | y = 14499x – 2721.1 |
| *Y. lipolytica* | Glucose | M7 | I_E_ | 0.9937 | y = 326.61x – 53.566 |
|  |  |  | A_E_ | 0.9991 | y = 15209x – 1833.4 |
|  |  |  | ф_fl_ | 0.9987 | y = 14330x – 2591.2 |
| *Y. lipolytica* | VFAs | M1 | I_E_ | 0.9936 | y = 326.66x + 14.426 |
|  |  |  | A_E_ | 0.9988 | y = 14776x – 239.84 |
|  |  |  | ф_fl_ | 0.9894 | y = 14622x – 1677.7 |
| *Y. lipolytica* | VFAs | M7 | I_E_ | 0.9992 | y = 326.76x – 34.023 |
|  |  |  | A_E_ | 0.983 | y = 14926x – 471.58 |
|  |  |  | ф_fl_ | 0.9971 | y = 14262x – 1068.4 |

*M1: R^2^ and standard curves equations obtained with *C. curvatum* and *Y. lipolytica* at month-1; *M7: R^2^ and standard curves equations obtained with *C. curvatum* and *Y. lipolytica* at month-7; *I_E_: fluorescence emission intensity; *A_E_: fluorescence emission area; *ф_fl_: fluorescence quantum yield.
